# The Banff 2022 Kidney Meeting Work Plan: Data-driven refinement of the Banff Classification for renal allografts

**DOI:** 10.1016/j.ajt.2023.10.031

**Published:** 2023-11-04

**Authors:** Candice Roufosse, Maarten Naesens, Mark Haas, Carmen Lefaucheur, Roslyn B. Mannon, Marjan Afrouzian, Nada Alachkar, Olivier Aubert, Serena M. Bagnasco, Ibrahim Batal, Chris O.C. Bellamy, Verena Broecker, Klemens Budde, Marian Clahsen-Van Groningen, Shana M. Coley, Lynn D. Cornell, Darshana Dadhania, Anthony J. Demetris, Gunilla Einecke, Alton B. Farris, Agnes B. Fogo, John Friedewald, Ian W. Gibson, Catherine Horsfield, Edmund Huang, Syed A. Husain, Annette M. Jackson, Jesper Kers, Željko Kikić, Amanda Klein, Nicolas Kozakowski, Helen Liapis, Massima Mangiola, Robert A. Montgomery, Brian Nankinvell, Desley A.H. Neil, Peter Nickerson, Marion Rabant, Parmjeet Randhawa, Leonardo V. Riella, Ivy Rosales, Virginie Royal, Ruth Sapir-Pichhadze, Pinaki Sarder, Minnie Sarwal, Carrie Schinstock, Mark Stegall, Kim Solez, Jeroen van der Laak, Chris Wiebe, Robert B. Colvin, Alexandre Loupy, Michael Mengel

**Affiliations:** 1 Department of Immunology and Inflammation, Faculty Medicine, Imperial College London, London, UK; 2 Department of Microbiology, Immunology and Transplantation, KU Leuven, Leuven, Belgium; 3 Department of Pathology and Laboratory Medicine, Cedars-Sinai Medical Center, Los Angeles, California, USA; 4 Université Paris Cité, INSERM, PARCC, Paris Institute for Transplantation and Organ Regeneration, France & Department of Nephrology and Transplantation, Saint-Louis Hospital, Paris, France; 5 Department of Internal Medicine, Division of Nephrology, University of Nebraska Medical Center, Omaha, Nebraska, USA; 6 Department of Pathology, University of Texas Medical Branch at Galveston, Texas, USA; 7 Department of Medicine, The Johns Hopkins University School of Medicine, Baltimore, Maryland, USA; 8 Université Paris Cité, INSERM, PARCC, Paris Institute for Transplantation and Organ Regeneration, France & Department of Transplantation, Necker Hospital, Paris, France; 9 Department of Pathology, Johns Hopkins School of Medicine, Baltimore, Maryland, USA; 10 Pathology & Cell Biology, Columbia University Irving Medical Center, New York, USA; 11 Division of Pathology, University of Edinburgh, Scotland; 12 Department of Clinical Pathology, Sahlgrenska University Hospital, Gothenburg, Sweden; 13 Department of Nephrology, Charite Universit atsmedizin, Berlin, Germany€; 14 Department of Pathology and Clinical Bioinformatics, Erasmus University Center Rotterdam, Rotterdam, Netherlands; 15 Institute of Experimental Medicine and Systems Biology, RWTH Aachen University, Aachen, Germany; 16 Transplant Translational Research, Arkana Laboratories, Arkansas, USA; 17 Department of Laboratory Medicine and Pathology, Mayo Clinic, Rochester, Minnesota, USA; 18 Department Medicine, Weill Cornell Medical College of Cornell University, New York, New York, USA; 19 UPMC Hepatic and Transplantation Pathology, Pittsburg, Pennsylvania, USA; 20 Department of Nephrology and Rheumatology, University Medical Center Gottingen, Germany€; 21 Department of Pathology and Laboratory Medicine, Emory University, USA; 22 Department of Pathology, Microbiology and Immunology, Vanderbilt University Medical Center, Nashville, Tennessee, USA; 23 Comprehensive Transplant Center, Northwestern University, USA; 24 Department of Pathology, University of Manitoba, Winnipeg, Canada; 25 Dept of Histopathology, Guy’s & St Thomas’ Hospital, London, UK; 26 Department of Medicine, Division of Nephrology, Cedars-Sinai Medical Center, Los Angeles, California, USA; 27 Division of Nephrology, Columbia University, New York, New York, USA; 28 Department of Surgery, Duke University, Durham, North Carolina, USA; 29 Department of Pathology, Leiden University Medical Center, Netherlands; 30 Department of Pathology, Amsterdam UMC, University of Amsterdam, Amsterdam, Netherlands; 31 Department of Urology, Medical University of Vienna, Vienna, Austria; 32 Critical Path Institute, Tucson, Arizona, USA; 33 Department of Pathology, Medical University of Vienna, Vienna, Austria; 34 Ludwig Maximillian University Munich, Nephrology Center, Germany; 35 Transplant Institute, NYU Langone Health, New York, USA; 36 NYU Langone Transplant Institute, New York, USA; 37 Department of Renal Medicine, Westmead Hospital, Westmead, New South Wales, Australia; 38 Department of Cellular Pathology, Queen Elizabeth Hospital Birmingham and Institute of Immunology and Immunotherapy, University of Birmingham, Birmingham, UK; 39 Department of Medicine and Department of Immunology, University of Manitoba, Winnipeg, Canada; 40 Pathology department, Necker-Enfants Malades Hospital, Paris, France; 41 Pathology, Thomas E. Starzl Transplant Institute, University of Pittsburgh, Pittsburgh, Pennsylvania, USA; 42 Department of Medicine, Massachusetts General Hospital, Harvard Medical School, Boston, Massachusetts, USA; 43 Immunopathology Research Laboratory, Department of Pathology, Massachusetts General Hospital, Boston, Massachusetts, USA; 44 Maisonneuve-Rosemont Hospital, University of Montreal, Quebec, Canada; 45 Division of Nephrology & Multiorgan Transplant Program, McGill University, Montreal, Quebec, Canada; 46 Department of Medicine-Quantitative Health, University of Florida College of Medicine, Florida, USA; 47 Division of MultiOrgan Transplantation, UCSF, San Francisco, California, USA; 48 Department of Internal Medicine, Division of Nephrology and Hypertension, Mayo Clinic, Rochester, Minnesota, USA; 49 Department Transplantation Surgery, Mayo Clinic, Rochester, Massachusetts, USA; 50 Department of Laboratory Medicine and Pathology, University of Alberta, Edmonton, Canada; 51 Department of Pathology, Radboud University Medical Center, Netherlands; 52 Department of Pathology, Massachusetts General Hospital, Harvard Medical School, Boston, Massachusetts, USA

**Keywords:** Banff, allograft, pathology, classification, working groups, work plan

## Abstract

The XVIth Banff Meeting for Allograft Pathology was held in Banff, Alberta, Canada, from September 19 to 23, 2022, as a joint meeting with the Canadian Society of Transplantation. In addition to a key focus on the impact of microvascular inflammation and biopsy-based transcript analysis on the Banff Classification, further sessions were devoted to other aspects of kidney transplant pathology, in particular T cell–mediated rejection, activity and chronicity indices, digital pathology, xenotransplantation, clinical trials, and surrogate endpoints. Although the output of these sessions has not led to any changes in the classification, the key role of Banff Working Groups in phrasing unanswered questions, and coordinating and disseminating results of investigations addressing these unanswered questions was emphasized. This paper summarizes the key Banff Meeting 2022 sessions not covered in the Banff Kidney Meeting 2022 Report paper and also provides an update on other Banff Working Group activities relevant to kidney allografts.

## 1. Preamble

The XVIth Banff Meeting for Allograft Pathology was held in Banff, Alberta, Canada, from September 19 to 23, 2022, as a joint meeting with the Canadian Society of Transplantation (CST). A wide spectrum of emerging issues and unmet needs in kidney transplant diagnostics was covered at the meeting, and a work plan for the currently unanswered questions was developed to address these in the coming years. The Banff 2022 Kidney Meeting Report, which is published as a separate manuscript, focuses on the diagnosis of cases with microvascular inflammation and antibody-mediated rejection (AMR), clinical adoption of biopsy-based transcript analysis, and their impact on the current Banff Classification.^1^ This Banff 2022 Kidney Meeting Work Plan summarizes progress reports, discussions, and subsequent work plans developed by the various Banff Working Groups (Tables 1–6; Supplementary Tables S1–S10), with more detail given for those topics that were covered in sessions and talks and widely commented on at the Banff 2022 meeting: T cell–mediated rejection, activity and chronicity indices, digital pathology and machine learning, xenotransplantation, and use of the Banff Classification in clinical trials and observational investigations. The Banff Working Group activities (Table 1) represent the scientific engine driving the constant data- and evidence-based refinement of the international Banff Classification for Allograft Pathology.

## 2. T cell–mediated rejection and the role of inflammation in areas of interstitial fibrosis and tubular atrophy (i-IFTA)

In a session on tubulointerstitial inflammation at the Banff 2022 meeting, 2 of the more problematic diagnoses in the Banff Classification —chronic active T cell–mediated rejection (caTCMR) and Borderline (suspicious) for acute TCMR—were critically appraised. In addition, a set of questions for further investigation was outlined by the TCMR Working Group (Table 2).

### 2.1. caTCMR

Tubulointerstitial (grade I) caTCMR was introduced in the Banff Classification in 2017 and updated in 2019, based on the strong association between i-IFTA and graft loss, and evidence of its association with underimmunosuppression and previous active TCMR.^2,3^ However, i-IFTA is noted not only in patients with previous TCMR but also in those with previous or concurrent pyelonephritis, polyomavirus-associated nephropathy, recurrent or de novo kidney diseases, and AMR. This nonspecificity of the i-IFTA lesion justified a definition of caTCMR that included (1) a requirement to exclude other diseases, and (2) a requirement for a minimum threshold of concurrent tubulitis and ti lesion. i-IFTA has to be considered along with the ti score, to avoid giving too much weight to very small areas of scarred cortex with heavy inflammation. Accordingly, it was acknowledged that the definitions for caTCMR need further clinical validation.

In a dedicated Banff 2022 session, the high prevalence of i-IFTA was highlighted (~50% of biopsies taken >1-year posttransplant),^4^ as well as its association with the temporal development of cv and cg lesions scores over time. The following further data supporting the association between caTCMR and poor outcomes were reported and discussed at the meeting.^5,6^ First, 8-year survival from the composite outcome (graft loss and doubling of serum creatinine) following a diagnosis of caTCMR made on a protocol biopsy (1-year posttransplant) is around 65%, and in an indication biopsy around 50%. Second, clinical response to treatment may be observed in a minority of cases of caTCMR (20%, reported^7^). However, some cases appear to show histological response, with a significant reduction in activity scores t, ti, and i-IFTA in posttreatment biopsies, without reduction in chronicity scores.^8^

Gene expression studies in bulk tissue revealed a different molecular profile related to inflammation in cases with caTCMR as opposed to the inflammation in cases of active TCMR, the latter showing predominant interferon-gamma pathway activation with acute kidney injury, and the former showing signals related to injury-repair and mast cells.^7,9^ In 1 study, more cases of i-IFTA had AMR-related transcriptomic signatures than TCMR-associated ones,^9^ and acute kidney injury-associated transcripts drove the poor outcomes in i-IFTA cases. In keeping with this observation there is a significant increase in patients with donor-specific antibodies (DSA) and/or C4d-positivity in patients with i-IFTA.^6^

Finally, previous changes in the definitions of tubular atrophy, t, and t-IFTA have led to confusion and likely inconsistent application of the definition of caTCMR, even among specialists. The definitions of these lesions were clarified and agreed upon (reported in the Banff 2022 Kidney Meeting Report).^1^

In summary, current evidence converges to suggest that i-IFTA is nonspecific both in its clinical associations and its molecular profile, but that it has a poor prognosis, even at a low i-IFTA = 1 score, and worse than fibrosis without inflammation. However, further data are needed to provide evidence that the Banff Classification thresholds for caTCMR (including t and ti thresholds in addition to i-IFTA) guarantee an increased diagnostic specificity for a T cell–mediated rejection process. Moreover, the effectiveness of therapeutic approaches for caTCMR has not been examined sufficiently, and it remains unclear whether the disease process and its association with graft failure can be halted. An alternative approach discussed at the Banff 2022 meeting would be to consider the i-IFTA lesion score as a diagnosis-agnostic prognostic feature only (see discussion below on activity and chronicity indices). Further investigations are needed to determine the diagnostic and prognostic value of i-IFTA in the Banff Classification, a focus of the TCMR Working Group (Table 2).

### 2.2. Borderline (suspicious) for acute TCMR (aTCMR)

The definition of borderline for TCMR (BL) arose during conversations at the time of merger of the Collaborative ClinicalTrialsin Transplantation (CCTT) classification with the Banff Classification. The threshold of the Banff i lesion score for TCMR at >25% established at these discussions during the 1990s is relevant to an era in which less effective immunosuppressive drugs were used, and thus may be too high. This threshold currently leads to a high number of cases being called BL based on active infiltrates that represent <25% of nonscarred interstitium. Recent evidence shows that a significant portion of BL behave as a TCMR.^10,11^ Patients with more HLA molecular mismatches have an increased risk of BL which—in the current era of immunosuppressive drugs—likely represents an alloimmune response in many cases.^11^ Patients with a first episode of BL or TCMR have a second episode in about 50% of cases, with this persistent or recurrent insult increasing the risk of graft loss.^12,13^

Studies have shown that BL is often treated in indication biopsies but inconsistently in protocol/surveillance biopsies, while no evidence-based standards for treatment of borderline and TCMR exist.^14^ As indicated at the Banff 2022 meeting, a re-evaluation of the aTCMR and BL definitions and thresholds for the current era of immunosuppression is needed.

## 3. AMR

Although the Banff 2022 Meeting Report^1^ proposes an approach for dealing with biopsies showing incomplete features of AMR, such as microvascular inflammation (MVI) that is DSA-negative and C4d-negative, or MVI below the threshold for a diagnosis of AMR, further investigation into the impact of these phenotypes is needed. The AMR Working Group will focus on this and related questions: Does DSA modify the risk of graft failure related to MVI? Is this risk modified by population characteristics? What are key population, assays, outcome definitions, and therapy variables that must be considered and reported on when designing studies geared toward reducing antibody-mediated injury? How can we optimize communication across the multidisciplinary team to make the AMR diagnosis? (Supplementary Table S1).

## 4. Biopsy-based transcript diagnostics

The Banff 2022 Kidney Meeting Report summarizes the output of discussions around the applicability of transcript analysis for the diagnosis of rejection.^1^ The Banff community felt that premature introduction of transcript-based diagnosis—which is not widely available and requires further validation—within the diagnostic classification, could create confusion among users of the classification and potentially lead to incorrect clinical decisions. For this reason, the wording in the classification “if thoroughly validated” was replaced by “if thoroughly validated for this context of use and available.”

In order to generate the evidence needed to justify the introduction of transcript-based diagnosis, the Biopsy-based Transcript Diagnostics (formerly “molecular diagnostics”) Working Group has formulated questions that need to be answered. Although both the MMDx^®^ platform and the Banff Human Organ Transplant (B-HOT) gene panel designed for the Nanostring nCounter platform (and indeed other methods of gene expression investigation such as RT-PCR or reverse transcriptase multiplex ligation-dependent probe amplification)^15,16^ can produce a multitude of different classifiers, it remains to be determined which classifier(s) perform(s) best for TCMR and for AMR diagnosis. Which thresholds should be used for clinical diagnosis and decision-making? Does addition of gene expression analysis significantly improve patient care and outcomes? How could an MMDx^®^ score convert into a B-HOT score and vice versa, to allow standardization of clinical decisions between centers using different platforms?^17^ Do biopsy-based transcript diagnostics offer similar or additional information to “activity and chronicity indices” provided by light microscopy? What is the clinical context of the use of biopsy-based transcript diagnostics? It was suggested to primarily implement molecular assessment in cases fulfilling some, but not all Banff criteria of AMR or TCMR (eg, BL and suspicious cases), or when there is a discrepancy between clinical/serological findings vs histology. What is the cost/benefit health-economic analysis of biopsy-based transcript diagnostics? What minimal number of transcripts needs to be measured? It is likely to be much less than what current platforms (MMDx^®^; B-HOT panel) provide. What is the predictive value of biopsy-based transcript diagnostics for effectively guiding therapy?

A proposed validation work plan including assessment of the context of use and clinical utility of biopsy-based transcript diagnostics was developed by the Biopsy-based Transcript Diagnostics (formerly “molecular diagnostics”) Working Group (Table 3).

## 5. Banff active/chronic lesion scores, activity/chronicity indices

In the current Banff Classification, the temporal disease dynamics are reflected in the active, chronic/active, and chronic subcategories of TCMR and AMR. The evaluation of kidney transplant biopsies, both histologically and using molecular tools, contains information on the disease stage and reversibility of disease processes. A distinction is made between “active Banff Lesion Scores” (i, t, v, g, ptc, C4d), “chronic Banff Lesion Scores” (ci, ct, cv, cg, ptcml), and as proposed in Banff 2019 also “active & chronic Banff scores” (ti, i-IFTA, t-IFTA, pvl). As outlined in Banff 2019^3^ and on the official Banff Classification website (https://banfffoundation.org/central-repository-for-banff-2019-resources-3/) inclusion of individual lesion scores in the biopsy report is advised. Conceptually, temporal links have been proposed between the active lesions leading to later chronic counterparts as the matrix is remodeled as a result of the inflammation. For instance, g associates with later cg, t and t-IFTA with later ct, i with later ci, v with later cv, and ptc with later ptcml.

The distinction between different disease stages (active, chronic/active, and chronic disease) is based on arbitrary thresholds. These thresholds have been the topic of heavy debate at past Banff meetings and were discussed again at Banff 2022 (see TCMR). At the Banff 2019 meeting (and later refined in a viewpoint paper), it was proposed that activity and chronicity indices could be used for cases of AMR,^3,18^ similar to what has been done for lupus nephritis.^19,20^ This approach with a calculated “chronicity index” based on Banff Lesion Scores was further explored and recently shown to be associated with graft failure in patients with AMR.^21^

Beyond application in the context of AMR, data from studies carried out in Leuven, Belgium, and validated in Paris and Lyon, France, were presented that made a case for extending the assessment of such activity and chronicity indices to all kidney transplant biopsies, independent of the disease entity (https://rejectionclass.eu.pythonanywhere.com). ^22,23^ These indices, calculated based on their relative association with graft failure, are strong prognostic factors for graft failure, and, therefore clinically meaningful. Moreover, such indices might provide clinicians with greater insight as to the severity of injury and its reversibility. High activity with low chronicity in an adequate biopsy sample may be more reversible than a biopsy with low activity/high chronicity. Activity and chronicity indices may therefore have predictive potential (ie, predicting which therapies could work, and which less), although this needs to be tested. The continuous nature of some of these indices would also allow longitudinal assessment of the dynamics of disease severity (activity/chronicity) more easily over time. Similar to chronic injury detected histologically, increased injury/damage-related transcripts are also associated with later graft loss in either TCMR or AMR.^24,25^

Despite the enthusiasm at the Banff 2019 and Banff 2022 meetings to adopt activity and chronicity indices, concern was also expressed that lesion scores and calculated indices might be erroneously equated with diagnosis. Clinical context (graft function in particular) is also important. In addition, individual lesion scores and activity/chronicity indices do not replace overall assessment of tissue for diagnoses other than rejection that also impact Banff Lesion Scores (eg, pyelonephritis, recurrent glomerulonephritis). In addition, it is important to recall that highly sensitized patients undergoing biopsies early posttransplant may show very “active” AMR with features like thrombotic microangiopathy or severe acute tubular injury, not captured in the activity index.^26^ In other words, it should be made clear that activity and chronicity indices do not replace diagnosis but add to it.

In summary, several published studies and the discussions held at the meeting strongly support use of activity and chronicity indices in reporting kidney transplant biopsies. However, the relation to the current Banff subcategories (eg, active, chronic/active, chronic) remains unclear. Ultimately, activity and chronicity indices might replace Banff subcategories and thus lead to significant changes in the classification, similar to the recently modified NIH classification for lupus nephritis.^20^ Moreover, at least 2 different scoring systems have been put forward so far.^21–23^ Therefore, it was decided to initiate a new Banff Working Group, with specific questions to answer with the goal of further validating activity and chronicity scoring in a clinical context (Table 4).

## 6. Digital transplant pathology and machine learning

Several sessions in the Banff 2022 meeting reviewed advances in the field of machine learning applied to digital pathology. The current work of the Banff Digital Pathology Working Group is the subject of 2 publications^27^(Farris et al, under review), and future plans are outlined in Supplementary Table S10. A growing number of kidney transplant pathologists are using digital pathology for their daily diagnostic practice, research, and clinical trials, facilitating accessibility, and sharing of digitized whole slide images (WSI), without apparent quality loss.^28^ Moreover, many are starting to curate datasets of WSI with or without annotations that can be used to train models to perform diagnostic tasks.

Potential deep learning–derived decision-support systems in kidney transplant pathology include applications that deliver segmentation of microanatomical structures (eg, glomeruli^29^), high-throughput morphometric quantification (eg, tubular diameters^30^), object detection (eg, inflammatory cells^31,32^), and weakly supervised slide-level classification (eg, rejection versus nonrejection to prioritize workload), with or without computer-assisted diagnosis overlays to WSI that guide the nephropathologist toward biopsy zones with highly informative features.^33^ Also, the evaluation of difficult lesions like transplant glomerulopathy could be improved with prognostic implications, as recently demonstrated.^34^ It has been shown that digital features significantly correlate with Banff Lesion Scores, but are also more sensitive to subtle pathological changes, below the thresholds in the Banff grading system.^35^ Composite damage scores calculated from those digital features outperformed Banff scores with superior graft loss prediction accuracy, indicating the potential of these models in addition to or beyond the Banff scoring system, eg, also when applied to time zero biopsies.^35^

A major bottleneck in developing deep learning models remains the access to large datasets of WSI annotated both for their pathology features and clinical features including outcomes. Methods such as the H-AI-L (Human Artificial Intelligence Loop) have facilitated algorithmic training with human input for annotations minimized.^36^ Algorithmic auditing by sensitivity analysis and stress-testing, supported byearlyadoption of AI-specific reporting guidelines (eg, DECIDE-AI^37^), has been highlighted to allow for fair and transparent algorithm development and implementation.

## 7. Xenotransplant pathology

A joint session with the CST covered progress in genetically modified pig-to-human xenotransplantation. Within this session, kidney xenograft pathology was covered, including both pig-to-primate experimental models,^38–40^ and early pig-to-human decedent temporary transplants.^41,42^ Some similarities in the pathology were noted comparing experimental models with first in human observations, in some (but not all) cases, glomerular fibrin and platelet thrombi and other features of endothelial injury were observed, while typical Banff rejection features were not represented. There is a potential for unique antibody-mediated pathology to appear over time, related to the myriad of antigenic differences in pigs and primates, and as grafts survive longer.

The B-HOT Nanostring panel of transcripts was considered for its potential to serve as a Banff Pig Organ Transplant panel, by analyzing gene homology, with early results documenting an increase in AMR-associated gene expression.^43^ However, a custom panel with a mix of pig selective and human/nonhuman primate selective genes might ultimately be needed. Recommendations for reporting of pathology in xenotransplantation are currently being considered and a proposal for a Banff Working Group for Xenotransplantation pathology was put forward to coordinate efforts (Table 5). Grading of lesions of thrombotic microangiopathy and C4d are central to biopsy evaluation, and immunostaining for immunoglobulin and complement, ultrastructural and RNA analysis (bulk and/or spatial) are recommended.

## 8. Banff classification for clinical trials

### 8.1. Histological endpoints

In a session dedicated to the use of the Banff Classification for clinical trials (both as trial endpoint and for definition of inclusion criteria) with input from the “Surrogate Endpoints Working Group,” it was concluded that the classification for clinical use should fully align with the definitions accepted for clinical trials. If histological endpoints are used as endpoints for clinical trials, there is consensus on the need to distinguish between AMR and TCMR and avoid the use of “biopsy-proven acute rejection,” as was outlined recently by a working group of the European Society of Organ Transplantation. The European Medicines Agency (EMA) agreed that the histological subtype of rejection, and the clinical context (indication vs protocol biopsy) are useful specifications and noted that this detailing might be very informative in profiling the efficacy of immunosuppression.^44–46^

It was also noted that for clinical trials, discontinuous/semiquantitative histologic scoring leads to difficult reproducibility. This is a major issue that leads to lower power of studies and the need for larger study groups and increasing study costs. It is therefore key to use well-defined phenotypes in a clinical trial. This requires primarily a clear diagnostic definition according to the most current Banff Classification, panels of (central) pathologists (3 optimal to avoid a tie), adjudication mechanisms in case of disagreements, WSI for centralized slide review, and auditable assessments (Table 6). In addition, it is hoped that the automated digital image analysis could help increase the reproducibility and estimation of the severity of kidney transplant injury. For now, none of these digital pathology algorithms is accepted as the primary endpoint definition for clinical trials.

A minimal set of variables, including clinical information, is needed to come to robust final diagnoses, as example, indicated in a recent study on eculizumab in the prevention of AMR, where there was a discrepancy between local and central pathology results (influencing the statistical analysis), due to an initial lack of clinical information such as DSA available to central pathologists.^47^

Although biopsy-based transcript diagnostics could hold promise for reproducible and quantitative assessment of biopsies, their added value, usefulness, and acceptability by health authorities as an endpoint for therapy registration trials need further discussion.

Finally, it is advised that future clinical trials do not solely collect and report the final Banff Diagnostic Categories (which are approved as primary endpoints in novel therapy registration trials^44–46,48^), but also the granular Banff Lesion Scores and Additional Diagnostic Parameters. This would allow one to retrospectively reassess trial results^49^ and to retrospectively apply multidimensional prognostication systems such as iBox^50,51^ to the trial results. Open reporting of these granular data and public access is therefore recommended.

### 8.2. Surrogate endpoints

Discussions also covered the fact that the short-term outcomes traditionally used for trials in kidney transplantation are not necessarily reflected in long-term success, given the complex and multifactorial causes of late graft failure.^52,53^ Multidimensional (surrogate) endpoints that capture the multifactorial causes of graft failure at an early stage are therefore desirable. Several have been proposed,^54^ but only the iBox algorithm, essentially a multivariable Cox proportional hazards model, is sufficiently validated and calibrated for this purpose at this time.^50^

The iBox Scoring System is a composite biomarker built on the following clinically relevant components: eGFR; proteinuria; presence of anti-HLA DSA; Banff Lesion Scores (IFTA grade, g + ptc, cg, and i + t scores). Inclusion of these histological lesion scores is, however, not always required for the prediction accuracy of the composite biomarker. In some contexts, using the Abbreviated iBox Scoring System (inclusion of only graft functional parameters and DSA) could be sufficient. However, in other specific situations, for example, in sensitized patients and in trials on AMR, the use of the Full iBox Scoring System adds further prediction accuracy.

Recently Critical Path Institute’s Therapeutics Consortium, a public-private partnership with the FDA and various transplant stakeholders (professional societies, academia, industry, diagnostics, and patients), submitted the iBox Scoring System for consideration as a novel endpoint to facilitate new therapeutic development through EMA’s qualification of novel methodologies for drug development. EMA qualified the iBox Scoring System as a secondary endpoint prognostic for death-censored allograft loss in kidney transplant recipients, to be used in clinical trials to support the evaluation of novel immunosuppressive therapy applications.^55^ In this specific context of use, the c-statistics for the derivation dataset were 0.809 (0.01 standard error) for the Full (with biopsy) and 0.803 (0.01 standard error) for the abbreviated (without biopsy) iBox Scoring System. Full iBox Scoring System (with biopsy), has an Area Under the Curve (AUC) of the Receiver Operating Characteristic (ROC) of 0.85 (0.02 standard error), while the Abbreviated iBox Scoring System (without biopsy) reaches a ROC AUC of 0.84 (0.02 standard error). Although EMA did not qualify the iBox Scoring System as a surrogate endpoint, EMA encourages further development of the scoring system targeting potential future qualification as a surrogate endpoint. Parallel to the discussions in Europe, the qualification of the iBox Scoring System as a reasonably likely surrogate endpoint to support FDA’s Accelerated Approval program is ongoing.

## 9. Conclusions

As indicated in a recent review, over the past 30 years, the Banff Classification for Renal Allograft Pathology^18^ has shown agility in adapting to advancing pathophysiological insights, changing clinical and regulatory contexts, and new diagnostic techniques. This agility remains vital. An integrated consensus process is followed to avoid overly frequent and minor changes to the classification and consequent difficulties in following the Banff rules in routine clinical care and in clinical trials.^1^ In this paper, we outlined the existing knowledge gaps with a view to stimulating efforts by the internationaltransplantcommunitytogeneratetherespectivedataneeded to find answers to key unanswered questions. Progress will be addressed at the next Banff 2024 meeting, which will be held in Paris, France, September 16–20, 2024.

## Supplementary Material

Supplementary data

## Figures and Tables

**Table 1:** Overview of the Banff working group leaders and intended impact on the (future) Banff classification. For details on the scope and ongoing activities of each Banff Working Group, see Supplementary Material.

Working group	Leaders	Intended impact on the (future) Banff Classification
TCMR	P. Randhawa M. Rabant M. Naesens V. Kung T. Muthukumar	Refinement of diagnosis criteria for Borderline, pure acute TCMR, and chronic active TCMR. Improved correlations between Banff Diagnostic Categories and clinical outcomes in patients with borderline, acute, and chronic active TCMR.
AMR (formerly “Sensitized”)	L. Cornell R. Sapir-Pichhadze S. Bagnasco C. Schinstock D. Dadhania	Provide a multidisciplinary perspective on the application of AMR/MVI diagnoses in the Banff Classification, and their impact on patient management.
Biopsy-based transcript diagnostics (formerly “molecular diagnostics”)	M. Mengel B. Colvin A. Loupy	Collaborative efforts through the International Consortium for Diagnostics & Outcome in Transplantation (ICDOT) are underway for multicentric analytical and clinical validation of the B-HOT panel with the aim to define its clinical utility.
HIV+/HIV+ renal transplants	S. Bagnasco	Confirm applicability of the Banff Classification in the HIV+ Donor to HIV+ Recipient transplant cohort.
Electron microscopy	C. Roufosse V. Broecker S. Coley V. Royal	Propose evidence-based, consensus-driven criteria for when and how to perform electron microscopy for diagnosis of rejection in transplant biopsies, and reproducible electron microscopy EM criteria for diagnosis and prognosis.
Thrombotic microangiopathy	M. Afrouzian H. Liapis N. Kozakowski	Propose minimum criteria for reporting thrombotic microangiopathy posttransplant and provide further guidance for integrating with clinical decision-making.
Recurrent glomerular disease	N. Alachkar S. Bagnasco	Propose evidence-based consensus on pathological features and features predictive of outcome for recurrent or de novo glomerulopathies, to be integrated into Banff guidelines for best practice in the diagnostic evaluation of recurrent disease.
Banff rules and dissemination	J. U. Becker C. Roufosse	Create a basis for transparent dissemination of the content of the Banff Classification, meeting the needs of the transplant community.
Digital pathology	K. Solez A. B. Farris	Translate the rapid advances in digital pathology and deep learning to potential clinical use in transplant pathology.
Peritubular capillaritis	I. W. Gibson Ž. Kikić N. Kozakowski	Potential change to methods of ptc lesion score evaluation (ptc “burden,” immunophenotyping, machine learning application, etc.).
Minimally invasive diagnostics	A. Jackson M. Clahsen-Van Groningen E. Huang	Evaluation of the potential of minimally invasive biomarkers for integration into the Banff classification for Renal Allograft Pathology.
New Banff working groups initiated at Banff 2022		
Time zero biopsies	O. Aubert S. Husain D. Neil P. Randhawa	Prepare consensus recommendations on specific situations in which biopsies should or should not be performed as a part of donor workup, and on best practice for improved reproducibility of reporting.
Activity and chronicity Indices	M. Haas M. Naesens S. Seshan	Evaluate the potential added value of using activity and chronicity scores in conjunction with or instead of the current (acute/active, chronic/active, and chronic/inactive) Banff disease subtypes.
Xenotransplantation pathology	I. Rosales I. Batal	Propose evidence-based criteria for grading and scoring kidney xenograft biopsies and develop best practice for applying immunostaining, transcriptomic analyses, and for correlating interpretation of xenograft biopsies with antipig antibody levels.
Suspended Banff Working Groups		
Surrogate endpoints (suspended)	A. Loupy M. Naesens	With the qualification of iBox by EMA and progress with the FDA, no further work is needed by this WG at this stage and the WG is suspended.

**Table 2:** TCMR Working Group: Questions to be answered.

Acute TCMR and borderline (suspicious) for acute TCMR:
• What would be the effect of revised criteria for borderline (suspicious) for acute TCMR, including elements of CCTT classification, on frequencies, associations, and outcomes of this category?
• What is the relevance of the v lesion in the context of tubulointerstitial inflammation and the pathophysiologic and clinical distinction between TCMR grades, and the therapeutic implications?
• Can we define histologic features that distinguish between clinically significant borderline changes and those that need therapeutic intervention (edema, tubular injury other than tubulitis, eosinophils, ongoing tubulitis in areas with IFTA, etc.)?
• What is the clinical course of pure TCMR presenting in conjunction with DSA-negative, C4d-negative, microvascular inflammation?
Chronic active TCMR (caTCMR):
• What is the reproducibility of i-IFTA & t-IFTA lesions, and can the variability in the scoring be reduced, eg, through creation of educational materials, and possibly an External Quality Assessment Program?
• What are the frequencies, associations, and outcomes of caTCMR; alone and in combination with borderline changes and acute TCMR grade 1 and 2?
• Should extent of tubular atrophy and interstitial fibrosis (ie, ct and ci scores) be considered in the definition of caTCMR alongside i-IFTA and ti?
• Can activity and chronicity indices be used in TCMR rather than or in addition to reporting of acute vs caTCMR
• What is the exact definition of additional diagnostic parameter “other known causes of i-IFTA should be ruled out?”
• What effect does diagnosis of caTCMR have on patient management, as opposed to just acute TCMR and borderline changes?
• Does caTCMR respond to the treatments used for aTCMR?
• Could lowering the Banff 2019 threshold for caTCMR (eg, the threshold for i-IFTA score) be helpful to identify cases that are currently passing through the cracks and getting the label of caTCMR only when advanced fibrosis has developed?

**Table 3:** Biopsy-based Transcript Diagnostics Working Group (formerly “molecular diagnostics”): Validation plan.

• Since no transcript has diagnostic specificity (similar to Banff histologic lesions), consensus thresholds for molecular classifiers and gene sets associated with Banff lesions and diagnosis need to be established and validated for defined clinical context(s) of use
• What is the Analytical Validity of different molecular assays?
○ Reproducibility and normalization/calibration studies are needed to make molecular results comparable between centers and sequential biopsies
○ Head-to-head comparisons of different assays on the same biopsy are needed to allow conversion of results from different platforms.
• What is the clinical validity of different molecular assays?
○ Comprehensive multicenter (not only biopsies from different centers, but assays run at different centers on the same biopsies) and multiplatform studies of well-annotated cohorts including the full spectrum of the diseases and controls (including native kidneys/recurrent diseases) need to be conducted.
• What is the clinical utility of different molecular assays?
○ Diagnostic vs prognostic vs theragnostic claims need to be validated in prospective or retrospective, randomized trials showing improved outcomes using molecular tests
○ Health-economic parameters need to be assessed to demonstrate value for money of molecular tests in kidney transplant care.

**Table 4:** Activity and Chronicity Indices Working Group: Questions to be answered.

• Which calculator(s) of the activity index (AI) and chronicity index (CI) is/are validated and proposed for inclusion in the Classification? Recently proposed AI and CI are validated^21–23^ but should be subjected to independent multicenter validation before being considered for inclusion in the Classification.
• Do we need disease-specific activity and chronicity indices, or are there potential indices that may be relevant for all Diagnostic Categories?
• For which Banff Diagnostic Categories are the activity and chronicity index informative? Recently proposed AI and CI^21^ are only applicable for AMR and not TCMR.
• How do activity and chronicity indices relate to the Banff subcategories (active; chronic/active; chronic)?
• Is there added value of the “active & chronic Banff scores” (ti, i-IFTA, t-IFTA) in the calculation of the activity and chronicity indices, either generally or within a specific Diagnostic Category?

**Table 5:** Xenotransplantation Working Group: Questions to be answered.

• Which pathologic patterns unique to xenograft biopsies require separate criteria for evaluation and scoring?
• Given the overlapping pathologic features of thrombotic microangiopathy and acute antibody-mediated rejection, what are useful clinical, laboratory, and other markers/methodologies that can improve diagnostic accuracy?
• How can we develop a standardized method for reporting antipig antibodies and apply it to diagnostic pathologic evaluation?

**Table 6:** Updated Banff recommendations on best practice for pathology and molecular endpoints in clinical trials.

A. Banff recommendations for use of the Banff classification in clinical trials
• The primary effort should focus on applying the most recent Banff classification in clinical trials
• Exact criteria used for inclusion in the trial, highlighting any deviations from the current official Banff Classification, should be clearly outlined in the study protocol and reporting
• The inclusion of biopsy-based transcript diagnostics, or not, in clinical trials should be stated in the trial protocol and in the reporting
• Open reporting of and public access to the granular study data (including individual Banff Lesions Scores) to allow for retrospective reassessment of study results is recommended
B. Banff recommendations on best practices for pathology endpoints in clinical trials
• Pathologists to participate in the design and choice of endpoints
• Panel of (central) pathologists (3 optimal to avoid a tie)
• Sufficient clinical information to (central) pathologists for correct diagnosis, including detailed information on DSA status for AMR diagnosis
• Adjudication mechanism (how discordance between pathologists is addressed)
• Whole slide digital images for centralized slide review
• Auditable assessments (scoring that can be reviewed and audited externally)
• Granular scoring and reporting of all Banff lesions, not only final Banff Diagnostic Category (consider using detailed phenotyping and lesion scoring for *secondary* endpoints)
• Quantitate changes (use of continuous scores and percentages rather than semiquantitative scoring)
• Centralized processing of ancillary testing, eg, immunohistochemistry (C4d; immunoglobulins and complement) and electron microscopy
C. Banff recommendations on best practices for molecular endpoints in clinical trials
• Molecular evaluation of kidney transplant biopsies can be considered for clinical trials, if available
• Large reference data sets should be well-annotated
• High reproducibility/replication of assays is required before use in clinical trials
• Pathogenesis-based transcript strategy appears useful and can be completed by classifier approaches (no single gene test is specific)
• Centralized testing advantageous for multicenter trials
• Proper methodological approaches are needed (for assay performance, data analysis, ...). This adds statistical power, potentially reducing sample size and costs
• Quality assurance is mandatory (interlaboratory, interplatform, and interassay reproducibility; development of standardized positive and negative controls and quantitative diagnostic reference standard)

## Abbreviations:

AMR: antibody-mediated rejection
B-HOT: Banff Human Organ Transplant gene panel
BL: borderline for T cell–mediated rejection
caTCMR: chronic active T cell–mediated rejection
DSA: donor-specific antibodies
EMA: European Medicines Agency
HLA: human leukocyte antigen
i-IFTA: Banff lesions score for inflammation in areas of interstitial fibrosis and tubular atrophy
MVI: microvascular inflammation
TCMR: T cell–mediated rejection
WSI: whole slide image
