# Supplementary material for: The Banff 2022 Kidney Meeting Work Plan: Data-driven refinement of the Banff Classification for renal allografts
Source: Am J Transplant. Author manuscript; Available in PMC 2024 May 29. (PMC11135910; doi:10.1016/j.ajt.2023.10.031)
Supplement: Supplementary data [file NIHMS1996340-supplement-Supplementary_data.docx]

**SUPPLEMENTARY TABLES: Progress Reports from existing Working Groups**

| **Table S1 - AMR Working Group** (formerly “sensitised”) **Summary** |
| --- |
| Chairs: L. Cornell, R. Sapir-Pichhadze, S. Bagnasco, C. Schinstock, D. Dadhania |
| Key Priorities   - Study the role of MVI with and without DSA to donor HLA for diagnosis of AMR; for prognosis; and for guiding management. - Multidisciplinary expertise and collaborations with other Banff working groups (e.g., non-invasive biomarkers, ptc, activity vs. chronicity) to review how the Banff Classification system impacts management. |
| Methods   - Organize multidisciplinary conference with key international stakeholders in the antibody-mediated injury domain - Initiate a multicenter retrospective cohort study |
| Results to date   - The Banff AMR definition^1^ is vulnerable to misinterpretation with potential patient management implications^2^ - The information required for AMR diagnoses (e.g., DSA, non-HLA antibodies, transcriptomics) may not be readily available to healthcare providers^3^ - There is significant heterogeneity in the transplantation literature regarding population characteristics, immunological risk assessment, outcome definitions and therapies applied for the management of antibody-mediated injury^4^ - Provided recommendations for a standard set of variables that should be reported on to inform immunological risks^4^ |
| Future Work   - Engage international stakeholders to develop consensus on a minimal set of standard variables that should be considered when designing studies on antibody-mediated injury. - Design and begin conducting a multicentre retrospective cohort study evaluating the prognostic implications of MVI with and without anti-HLA DSA on graft outcomes in a diverse population of varied immunological risk. |
| Intended impact on Banff Classification:   - Multidisciplinary perspective on the application of AMR diagnoses and how they inform management. |

| **Table S2. HIV+/HIV+ renal transplants** |
| --- |
| Chairs: Serena Bagnasco; on behalf of the HOPE in Action consortium |
| Key Priorities  Investigate outcomes and pathology of HIV+ patients post-transplant comparing HIV Donor+/Recipient+ to HIV Donor-/Recipient+ Kidney Transplants |
| Methods   - Multicenter pilot study directly comparing HIV Donor+/Recipient+ to HIV Donor-/Recipient+ Kidney Transplants, n=75 HIV+ kidney transplants. Study completed - NIAID U01 trial “HOPE in Action: A Clinical Trial of HIV-to-HIV Deceased Donor Kidney Transplantation” – 2017-2022, PIs: Christine Durand and Dorry Segev, JHU. Study completed, analysis of the results ongoing |
| Results to date   - Pilot study: Transplant and HIV outcomes were excellent in both groups with a trend toward higher rejection in Donor+ cases^5^ |
| Future Work   - NIAID U01 trial: biopsy analysis on-going on 127/200 patients recruited. - Grant application for funding for longer follow up and further investigations submitted to NIH |
| Intended impact on Banff Classification:  Confirm applicability of the Banff classification in the HIV+ Donor to HIV+ Recipient transplant cohort |

| **Table S3. Electron Microscopy (EM)** |
| --- |
| Chairs: Verena Broecker, Shana Coley, Candice Roufosse, Virginie Royal |
| Key Priorities  Evidence base for diagnostic and prognostic ultrastructural parameters of rejection   - Establish reproducibility of ultrastructural glomerular and peritubular capillary (PTC) endothelial and basement membrane features and simplify scoring - Analysis of diagnostic associations of reproducible features with clinical, serological, and histological parameters - Establish cut-off values of ultrastructural glomerular and PTC endothelial and basement membrane features that are diagnostic for active and or chronic active AMR and/or predictive of poor outcomes - Establish whether the addition of the ultrastructural findings to the clinical, serological, and light microscopic features adds value for treatment decisions or for outcome prediction |
| Methods   - Multicentre investigation of ultrastructural features in kidney transplant biopsies from patients with and without antibodies against the graft and with a range of histological findings on light microscopy. |
| Results to date   - Methods for EM examination of kidney transplant biopsies were agreed and published in the Banff 2019 report^1^ |
| Future Work   - Work over the next 2 years will focus on the Key priority above |
| Intended impact on Banff Classification:  Evidence-based proposal for consensus on criteria for when and how to perform EM for diagnosis of rejection in transplant biopsies, and reproducible EM criteria for diagnosis and prognosis |

| **Table S4. Thrombotic microangiopathy (TMA)** |
| --- |
| Chairs: Marjan Afrouzian, Helen Liapis, Nicolas Kozakowski |
| Key Priorities:  Establish minimum criteria for the diagnosis of TMA in the renal allograft. |
| Methods:   - Delphi method of consensus generation, in 3 phases: Phase I, to produce consensus among pathologists; Phase II to create consensus among nephrologists; and Phase III (“consensus of the consensus groups”) to generate consensus among nephropathologists and nephrologists. |
| Results to date:   - Phase I (2016 – 2021): 9 Delphi rounds through which consensus was obtained (23 participants/ panelists). 5 rounds to establish criteria; 2 validation rounds through on-line, iterative and retrospective examination of 37 cases with or without TMA; final consensus on 11 light, 3 immunofluorescence and 4 electron microscopic criteria, and 2 clinical and 4 laboratory criteria^6,7^ - Phase II (2020 – ongoing): 31 nephrologists. 5 Delphi rounds completed and consensus obtained on: 1. criteria for diagnosis of Systemic TMA, 2. criteria for diagnosis of Localized TMA; 3. the most important etiologies and differential diagnoses that need to be entertained; 4. Treatment options of complex cases of TMA when it is associated with other events in the allograft; and 5. Nephrologists’ clinical recommendations. |
| Future work:   - Complete Phase II (clinical decision making): 1 validation round and 3 additional Delphi rounds - Phase III: “consensus of the consensus groups” |
| Intended impact on Banff Classification:   - Recommendations for minimum criteria for reporting TMA post-transplant: manuscript under review; projected presentation to Banff community 2024 - Phases II and III will provide further criteria for integrating with clinical decision-making |

| **Table S5. Recurrent glomerular disease** |
| --- |
| Chairs: Nada Alachkar, Serena Bagnasco |
| Key Priorities   - Establish pathologic guidelines for reporting of recurrent/de novo glomerular diseases (including FSGS, IgA nephropathy, membranous nephropathy, MPGN/C3GN) - Establish frequencies, clinical manifestations, and pathologic characteristics of recurrent/de novo glomerular diseases - Establish predictors of outcomes - Investigate co-occurrence of recurrent glomerular diseases with rejection and other transplant-associated lesions |
| Methods   - Biopsy specimens and clinical data collected from 10 international centers to create a posttransplant GN registry. - Correlation analysis and outcome analysis |
| Results to date  Completed study of 171 transplant recipients with end stage kidney disease (ESKD) due to IgAN; 100 of them with biopsy-proven recurrent IgAN (57 with complete MEST-C scores); 71 with no recurrence. Findings may validate the prognostic usefulness of the modified Oxford classification for recurrent IgAN and support the inclusion of the MEST-C score in allograft biopsy diagnostic reports.  Paper “Evaluation of the modified Oxford Score in Recurrent IgA Nephropathy in North American Kidney Transplant Recipients: The Banff Recurrent Glomerulonephritis Working Group Report” published in Transplantation.^8^ |
| Future Work   - In progress: completing data collection of FSGS recurrence; Data analysis and manuscript writing - Data collection of membranous nephropathy |
| Intended impact on Banff Classification:   - Proposal to use MEST-C score when evaluating IgA recurrence as prognostic indicator of kidney allograft survival at next Banff meeting - Pathological features for diagnosis and prognosis in other recurrent (and de novo) glomerular diseases investigated for integration in Banff guidelines/classifications |

| **Table S6. Rules and Dissemination Working Group** |
| --- |
| Chairs: Jan Becker, Candice Roufosse |
| Key Priorities   Creating a basis for transparent dissemination of the Banff content. |
| Methods   - Collation of up-to-date Banff Classification content for Kidney - Creation of a web repository for the up-to-date Banff content with manual of operation for updates - Survey among the transplant community about dissemination needs |
| Results to date   - Web repository for Banff kidney content 2019 online ([https://banfffoundation.org/central-repository-for-banff-2019-resources-3/](about:blank)). - Proposed manual of operation for Banff web repository updates and archiving submitted to the Banff Board of Directors |
| Future Work   - Survey of Banff dissemination needs among transplantation community and if needed, create a Banff User Group, to replace the Rules and Dissemination Group |
| Intended impact on Banff Classification:  Facilitate routine use of the Classification by creating a single, accessible, and clear repository for up-to-date Banff content. |

| **Table S7. Peritubular capillaritis (ptc) working group** |
| --- |
| Chairs: Nicolas Kozakowski, Ian Gibson, Željko Kikić |
| Key Priorities  Re-evaluation of scoring methods and thresholds for the ptc Lesion Score, across all Banff Diagnostic Categories |
| Methods   - International multicentric cohort for scoring single features of ptc (intensity/score, extent, cellular composition, dilation) - 17 centres participating. - Lesional constellations (for instance diffuse and low-grade ptc) categorized into a "ptc burden" - Correlation with disease activity, molecular findings and outcome, in AMR, TCMR, Borderline, mixed rejection patterns. - Immunophenotype of infiltrating peritubular capillary cells - Collaborate with the Banff Working Group for Digital Pathology and DIAGGRAFT group to apply AI to investigation of ptc lesion score |
| Results to date   - Preliminary unpublished - Capillaritis is more often monomorphic and focal in TCMR, and polymorphic and diffuse with larger mononuclear cells in AMR - Immunohistochemical typing shows a predominance of T cells (CD3-positive) in TCMR and a mix of T cells with monocytes (CD68-positive) in AMR - Dilation of peritubular capillaries is noted in AMR but not TCMR but is hard to quantify; segmentation of capillaries followed by automated measurement using machine learning may help - Early post-transplant acute tubular injury is associated with polymorphic "isolated ptc" that is in most cases C4d-negative, and may be accompanied by a v1 lesion; molecular analysis of a few individual cases does not show a diagnostic AMR signature - Initial correlation between ptc and molecular gene sets using Nanostring nCounter Technology in patients with chronic AMR (Eder et al, manuscript in preparation) |
| Future Work   - Reproducibility study for ptc scoring on n=50 cases - Correlation and outcome investigations on a larger multicenter cohort |
| Intended impact on Banff Classification:   - Proposal of change to methods of ptc lesion score evaluation (ptc “burden”, immunophenotyping, machine learning application etc) - Proposal of change to diagnostic categories for which ptc lesion score is relevant (currently only AMR) |

| **Table S8. Minimally Invasive Diagnostics working group** |
| --- |
| Chairs: Edmund Huang, Annette Jackson, Marian Clahsen-van Groningen |
| Key priorities  Evaluate the ability of commercially available and emerging urine and blood biomarkers considered in addition to biopsy evaluation to improve the diagnostic accuracy |
| Methods   - Review of literature - urinary transcriptomics, dd-cfDNA and urinary chemokines - We considered that a diagnostic biomarker should: (1) differentiate rejection from the absence of rejection; (2) be specific for rejection; (3) hold the potential to replace biopsies or, at minimum, have additive value over histology alone; and if possible (4) have prognostic value. |
| Results to date:   - The negative predictive value for individual biomarkers to rule-out rejection shows promise, however the positive predictive value remains low and limits their use as diagnostic tests. - Combinations of these biomarkers in addition to clinical parameters are being assessed as a means of improving specificity and allowing the detection of sub-clinical acute rejection. - Review of the literature published in *Transplantation*^9^ |
| Future Work:  Evaluate future studies that assess minimally invasive biomarkers in combination with kidney biopsy evaluation to more precisely classify allograft pathology. |
| Intended impact on Banff Classification:  None at this time |

| **Table S9. Questions to be answered by Time zero (pre + post-implantation) Biopsy Re-visited: a new (revived) WG** |
| --- |
| Chairs: Olivier Aubert, Syed Husain, Desley Neil and Parmjeet Randhawa |
| No centers in France and very few in Europe perform pre-implantation biopsies. In contrast, over half of recovered kidneys are biopsied in the U.S. with considerable heterogeneity in biopsy criteria and technique, and a much higher organ discard rate (~ 20% vs 10%), with overall comparable graft survival in Europe and the USA. Centers avoiding biopsies have concerns about increasing cold ischemia time, suboptimal reproducibility of non-expert readings, and conflicting literature on its utility. Biopsies are cited as the commonest reason for organ discard in the USA, although kidneys undergoing biopsy are frequently those with other concerns which trigger a biopsy, which then contributes to a decision to discard.   - Can we reach a consensus on specific clinical settings where implantation biopsy could be potentially useful? - What are potential solutions to suboptimal reproducibility e.g. promoting more uniform biopsy technique, use of rapid processing protocols, enabling telepathology readings by expert pathologists, machine learning? - Are the current thresholds used for discard in glomerular, tubulointerstitial and vascular compartments adequate? - Could visual aids for the various biopsy grading systems help with understanding and reproducibility? |
| Intended impact on Banff Classification:   - Propose recommendations on specific situations in which biopsies should or should not be performed as a part of donor work for reporting - Propose best practice for improved reproducibility - Propose thresholds and visual aids |

| **Table S10. Digital Pathology Working Group** **Summary** |
| --- |
| Chairs:   Brad Farris, Kim Solez |
| Key Priorities   - Image bank(s)/collection(s) - Algorithm sharing platform(s) - International competitions and/or trials to develop and test the best algorithms for given tasks |
| Methods   - Image bank(s)/collection(s): Pilot image bank: DPLab, https://dplab.gsu.edu/ & https://github.com/jkonglab/DigitalPathology - Mulitcenter-multinational DIAGGRAFT image challenge coordinated by Radboud UMC, Nijmegen, The Netherlands [https://www.computationalpathologygroup.eu/projects/diaggraft/](about:blank) - Eventually providing a benchmarking dataset for algorithmic challenges |
| Results to date   - Publication of Working Group goals^10^; second manuscript in preparation |
| Future Work   - DIAGGRAFT Study mentioned above - Communication/Networking for future collaborative efforts |
| Intended impact on Banff Classification:   - Image bank(s)/collection(s) to provide benchmark datasets for algorithmic challenges - Disseminate/publish plans for the process of algorithm development - Present developed algorithms to the Banff Community - Consider and discuss Regulatory approval process |

**REFERENCES TO THE SUPPLEMENTARY TABLES**

1. Loupy A, Haas M, Roufosse C, et al. The Banff 2019 Kidney Meeting Report (I): Updates on and clarification of criteria for T cell- and antibody-mediated rejection. Am J Transplant 2020;20(9):2318-2331. DOI: 10.1111/ajt.15898.

2. Schinstock CA, Sapir-Pichhadze R, Naesens M, et al. Banff survey on antibody-mediated rejection clinical practices in kidney transplantation: Diagnostic misinterpretation has potential therapeutic implications. Am J Transplant 2018. DOI: 10.1111/ajt.14979.

3. Schinstock CA, Askar M, Bagnasco SM, et al. A 2020 Banff Antibody-mediatedInjury Working Group examination of international practices for diagnosing antibody-mediated rejection in kidney transplantation - a cohort study. Transpl Int 2021;34(3):488-498. DOI: 10.1111/tri.13813.

4. Jatana SS, Zhao H, Bow LM, et al. Seeking Standardized Definitions for HLA-incompatible Kidney Transplants: A Systematic Review. Transplantation 2023;107(1):231-253. DOI: 10.1097/TP.0000000000004262.

5. Durand CM, Zhang W, Brown DM, et al. A prospective multicenter pilot study of HIV-positive deceased donor to HIV-positive recipient kidney transplantation: HOPE in action. Am J Transplant 2021;21(5):1754-1764. DOI: 10.1111/ajt.16205.

6. Afrouzian M, Kozakowski N, Liapis H, et al. Thrombotic microangiopathy in the renal allograft: results of the TMA Banff Working Group consensus on pathologic diagnostic criteria. Transpl Int 2023;In press.

7. Afrouzian M, Kozakowski N, Liapis H, et al. Delphi: a democratic and cost-effective method of consensus generation in transplantation. Transpl Int 2023;In press.

8. Alachkar N, Delsante M, Greenberg RS, et al. Evaluation of the Modified Oxford Score in Recurrent IgA Nephropathy in North American Kidney Transplant Recipients: The Banff Recurrent Glomerulonephritis Working Group Report. Transplantation 2023. DOI: 10.1097/TP.0000000000004640.

9. Huang E, Mengel M, Clahsen-van Groningen MC, Jackson AM. Diagnostic Potential of Minimally Invasive Biomarkers: A Biopsy-centered Viewpoint From the Banff Minimally Invasive Diagnostics Working Group. Transplantation 2023;107(1):45-52. DOI: 10.1097/TP.0000000000004339.

10. Farris AB, Moghe I, Wu S, et al. Banff Digital Pathology Working Group: Going digital in transplant pathology. Am J Transplant 2020;20(9):2392-2399. DOI: 10.1111/ajt.15850.
